# 晚期肺癌患者免疫功能的系统评价及临床意义

**DOI:** 10.3779/j.issn.1009-3419.2010.04.12

**Published:** 2010-04-20

**Authors:** 图雅 乌兰, 士勇 王, 微丽 杜, 晖 张, 远 张, 雪 曾, 飒 刘, 艳萍 刘, 璐 张, 哲 张, 英 何, 佳玲 王, 秀艳 武

**Affiliations:** 110032 沈阳，中国医科大学附属第四医院生物治疗科 Department of Biotherapy, the Fourth Affiliated Hospital of China Medical University, Shenyang 110032, China

**Keywords:** 肺肿瘤, 细胞免疫, 体液免疫, 调节性T细胞, 细胞因子, Lung neoplasms, Cell immunity, Humoral immunity, Regulatary T cells, Cytokine

## Abstract

**背景与目的:**

真实评价免疫功能对明确肿瘤的发生发展及给予适时治疗具有重要意义。本研究旨在系统评价晚期肺癌患者免疫功能及意义。

**方法:**

计数晚期肺癌患者和健康人外周血免疫细胞数；用流式细胞仪测定免疫细胞亚群比例和细胞内IL-4、INF-γ及穿孔素、颗粒酶水平；用MTT法评价淋巴细胞对肿瘤细胞株的抑制率及其增殖活性。

**结果:**

晚期肺癌患者组T、B、NK细胞绝对数及亚群比例均显著低于健康组（*P* < 0.05）；但患者组的调节性T细胞（4.00±1.84）%明显高于健康组（1.27±0.78）%（*P* < 0.05）。患者组的CD8^+^T细胞中IFN-γ、穿孔素及颗粒酶阳性率均显著低于健康组（*P* < 0.05）；而IL-4正好相反。患者组的免疫细胞增殖能力、PPD阳性率及对瘤细胞株的抑制率显著低于健康组（*P* < 0.05）。

**结论:**

晚期肺癌患者免疫功能较健康人明显低下。

## Abstract (en)

**Background and objective:**

The actual evaluation of immunological function is significant for studing the tumor development and devising a treatment in time. The aim of this study is to evaluate the immunological function of advanced lung cancer patients systematically, and to discuss the clinical significance.

**Methods:**

The nucleated cell amounts of advanced lung cancer patients and the healthy individuals were counted. The immune cell subsets and the levels of IL-4, INF-γ, perforin and granzyme in CD8^+^T cells by the flow cytometry were measured. The proliferation activity and the inhibition ratio of immune cells to several tumor cell lines were evaluated by MTT assay.

**Results:**

The absolute amounts and subsets of T, B, NK cells of advanced lung cancer patients were lower than the healthy individuals (*P* < 0.05); However, the proportion of regulatory T cells of advanced lung cancer patients (4.00±1.84)% was lower than the healthy individuals (1.27±0.78)% (*P* < 0.05). The positive rates of IFN-γ perforin, granzyme in CD8^+^T cells decreased while them in IL-4 did not in the advanced lung cancer patients compared to the healthy control group (*P* < 0.05). The proliferation activity of immune cells, the positive rate of PPD masculine and the inhibition ratio to tumor cells in the advanced lung cancer patients was lower than the healthy subsets obviously (*P* < 0.05).

**Conclusion:**

There was a significant immune depression in the advanced lung cancer patients compared to the healthy individuals.

免疫系统常常被赋予两种截然不同的功能，对外来感染性病原体等表现出强烈的免疫应答和排斥反应，而对自身抗原表现出完全的无反应或忽视。那么，对源于自身的肿瘤细胞，免疫系统应该表现出怎样的反应？何以在看似正常的个体中产生肿瘤？70年代提出的免疫监视学说不能很好地解释这种对立的现象。新近的免疫编辑学说认为，肿瘤免疫可分为3个阶段：免疫清除、免疫平衡和免疫逃逸。在肿瘤发展的早期，只能对那些免疫原性强的肿瘤细胞表现出清除作用，同时“筛选出”免疫原弱的肿瘤细胞，使其成为肿瘤的优势细胞，进一步增殖，达到免疫平衡、免疫逃逸。在后期的阶段，肿瘤细胞通过分泌细胞因子，直接抑制机体的免疫功能; 或者，诱导产生如调节性T细胞等抑制性细胞^[1, 2]^。因此，如何全面、真实地反映免疫功能依然是难题^[3]^。本研究选择晚期肺癌患者，除了研究T、NK、B细胞外，也测定了调节性T细胞，并结合体外的杀瘤活性、机制以及PPD等体内检测，以期能系统地评价患者的免疫功能，为适时的临床干预提供依据。

## 1 材料和方法

### 1.1 研究对象

收集2009年1月-2009年6月中国医科大学附属第四医院住院的晚期肺癌患者40例，其中男性20例，女性20例，所有病人均经病理组织学或细胞学检查确诊，其中鳞癌5例，腺癌30例，腺鳞癌3例，小细胞肺癌2例。根据AJCC第6版肺癌临床分期标准进行分期，Ⅲ、Ⅳ期判定为晚期，年龄46岁-67岁，平均年龄（57.5±9.3）岁。所有病例均无糖尿病、严重感染及其它与自身免疫相关疾病，且3个月以内未接受过化、放疗及免疫治疗。健康人对照组为中国医科大学附属第四医院体检中心的健康志愿者20例，男性10例，女性10例，年龄43岁-61岁，平均年龄（53.4±5.5）岁。

### 1.2 主要仪器和试剂

流式细胞仪为美国BD公司生产，型号FC-500-MPL。抗CD3-FITC/CD4-APC/CD8-PE/CD45-PerCP单克隆抗体和抗CD19-APC/CD3-FITC/CD16-PECD56-PE/CD45-PerCP单克隆抗体四色试剂盒、抗CD4-FITC/CD25-PE/FOXP3-APC单克隆抗体试剂盒、IFN-γ/IL-4检测用试剂盒、FITC标记的穿孔素、PE标记的颗粒酶荧光抗体、佛波酯、离子霉素、莫能霉素、破膜剂、溶血素均购自美国BD公司。CO_2_培养箱，酶标仪为Anthos公司生产，型号Anthos 2010 SW-Version 1.8。

### 1.3 方法

#### 1.3.1 免疫细胞计数

取病人及健康人外周肝素抗凝血2mL，采用SysmexK-21全自动血细胞分析仪进行血常规测定。

#### 1.3.2 体液免疫测定

取病人及健康人外周肝素抗凝血2mL，静置，4 000 rpm离心10 min分离血清，用散射浊光计免疫法测定血清免疫球蛋白和补体水平。

#### 1.3.3 外周血T、B、NK细胞比例测定

用流式细胞术测定，具体方法如下，各取外周肝素抗凝全血50 μL加入两个流式管中，分别加入抗CD3-FITC/CD4-APC/CD8-PE/CD45-PerCP、抗CD19-APC/CD3-FITC/CD16-PE CD56-PE/CD45-PerCP荧光抗体各20 μL，混匀，避光静置20 min后各管加入溶血素450 μL，混匀，避光静置15 min，用流式细胞仪分析T、B、NK细胞在全血细胞中所占的比例。

#### 1.3.4 调节性T细胞测定

用流式细胞术测定，具体方法如下，取病人及健康人外周肝素抗凝血各2 mL，分离外周血单个核细胞，调整细胞浓度为1×10^7^/mL，取100 μL加入抗CD4-FITC/CD25-PE荧光抗体20 μL，4 ℃避光静置30min，加Flow cytometry staining buffer（流式细胞染色液）2mL洗涤，离心5 min，弃上清，加固定/透膜剂1 mL，4 ℃避光静置30 min，加破膜剂2 mL，离心5 min，弃上清; 加抗FOXP3-APC荧光抗体20 μL，4 ℃避光静置30 min，加破膜剂2 mL洗涤，离心5 min，弃上清，加流式细胞染色液2 mL洗涤两次，加0.5 mL细胞染色液重悬细胞混匀，用流式细胞仪分析调节性T细胞比例。

#### 1.3.5 细胞内细胞因子及穿孔素及颗粒酶测定

用流式细胞术测定，具体方法如下，取病人及健康人外周肝素抗凝血各500 μL，加入RPMI-1640培养液500 μL、莫能霉素5 μL（1 mg/mL）、佛波酯20 μL（1 μg/mL）、离子霉素20 μL（20 μg/mL），37 ℃温度下5%CO_2_培养箱培养4 h，PBS洗涤两遍，离心5 min，小心吸弃上清，混匀; 取样500 μL，加CD3-PerCP、CD8-APC各20 μL，室温避光孵育15 min，加溶血素2 mL，室温避光孵育10 min，离心5 min，弃上清，加FACSLysis（通透剂）0.5 mL，室温避光孵育10 min，加含0.5%BSA的PBS液2 mL，离心5 min，弃上清; 加IFN-γ/IL-4试剂盒抗体20 μL或FITC-抗穿孔素抗体、PE-抗颗粒酶抗体试剂各20 μL，室温避光孵育30 min，加PBS（含0.5%BSA）2 mL洗涤一次，后离心5min，弃上清，加PBS 0.5 mL重悬细胞，用流式细胞仪分析细胞内细胞因子及穿孔素及颗粒酶表达水平。

#### 1.3.6 体外细胞毒性实验

用MTT法测定，具体方法如下：靶细胞（肺癌细胞株A549及胃癌细胞株GM823均购于上海中科院细胞库）用含10%胎牛血清的RPMI-1640培养液，于37 ℃、5%CO_2_培养箱中孵育，用胰酶消化传代，选对数生长期的肿瘤细胞，调整细胞浓度为8×10^4^/mL，加入96孔板中，每孔100 μL，37 ℃、5 %CO_2_培养箱中培养4 h贴壁生长。将病人及健康人外周静脉血单个核细胞作为效应细胞（方法同1.3.4），按效:靶比20:1加入，共同培养48 h，每孔加入5 mg/mL MTT溶液20 μL后继续培养4 h。后离心5 min，小心吸弃上清，每孔加入150 μL DMSO溶液震荡混匀，酶标仪测定每孔570 nm光密度值（optical density, OD），计算抑制率。抑制率（%）=1-（杀伤组OD值-效应细胞对照组OD值）/肿瘤细胞对照组OD值×100%。

#### 1.3.7 体内细胞毒性试验

用结核菌素试验方法测定，即将PPD（人型结核菌素纯蛋白衍生物）0.1 mL注射到人体前臂曲侧中上部1/3处皮内，观察72 h反应，以硬结小作为判断反应的标准：无硬结或硬结直径≤4 mm为阴性; 5 mm-9 mm为弱阳性; 10 mm-19 mm为阳性; ≥20 mm或虽 < 20 mm但局部出现水泡和淋巴管炎等均为强阳性。

#### 1.3.8 细胞增殖测定

取病人及健康人外周静脉肝素抗凝血5 mL，通过淋巴细胞分离液分离单个核细胞，加入含10%胎牛血清的RPMI-1640培养液，调整细胞浓度为1×10^7^/mL，加入抗CD3抗体50 μL，共培养4 d，每24 h用0.4%台盼蓝染色记数活细胞，以时间为横坐标，细胞数为纵坐标绘制细胞生长曲线。

### 1.4 统计学处理

实验数据用SPSS 11.0统计软件分析，检测结果以Mean±SD表示，除体内细胞毒性试验结果进行χ^2^检验外，其它数据均采用*t*检验，*P* < 0.05表示差异有统计学意义。

## 2 结果

### 2.1 免疫细胞绝对计数的比较

20例晚期肺癌患者外周血白细胞总数及淋巴细胞、单核细胞绝对数明显低于健康对照组，两者有统计学差异（*t*分别为6.49、6.05、4.80，*P*分别为0.006、0.028、0.046），而晚期肺癌患者外周血中性粒细胞绝对数与健康对照组无统计学差异（*t*=1.67, *P*=0.217），结果见图 1。

**1:**
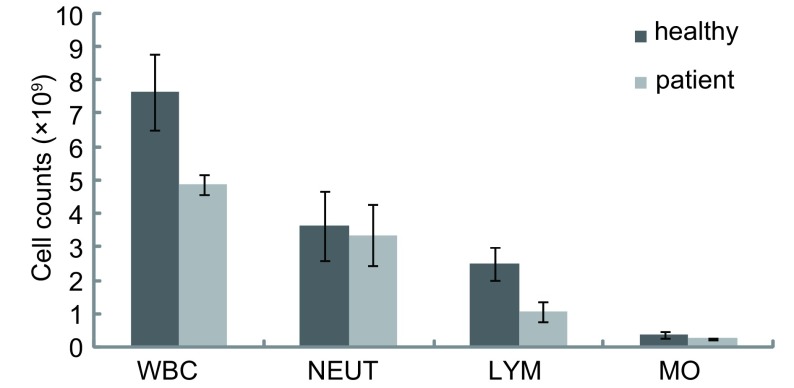
正常人及晚期肺癌患者外周血免疫细胞绝对数的比较（×10^9^） Comparisons of the absolute nucleated cell counts between advanced stage of lung cancer patients and healthy individuals (×10^9^)

### 2.2 体液免疫各项指标的比较

20例晚期肺癌患者外周血中，IgG、IgA水平较健康对照组有所升高，但无统计学差异（*P*分别为0.058、0.086）; IgM水平明显低于健康对照组（*P*=0.003）; 补体C3及C4水平均明显高于健康对照组（*P*分别为0.012、0.004），结果见图 2。

**2:**
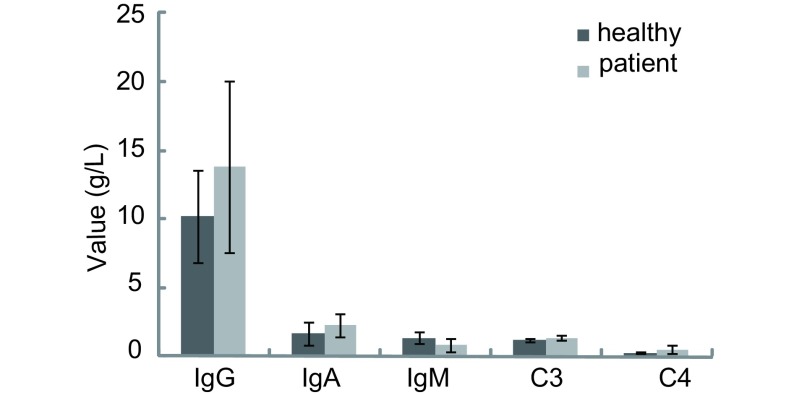
正常人及晚期肺癌患者体液免疫水平比较（g/L） Comparisons of the humoral immunity between advanced stage of lung cancer patients and healthy individuals (g/L)

### 2.3 外周血中T、B、NK和调节性T细胞比例的比较

20例晚期肺癌患者外周血中B细胞（CD19^+^）及NK细胞（CD3-CD16^+^CD56^+^）百分比显著低于健康对照组（*t*分别为6.50、4.73，*P*分别为0.017、0.038）; 晚期肺癌患者CD3^+^T细胞百分比显著低于健康对照组（*t*=8.75, *P*=0.006）; 患者CD4^+^T细胞百分比及CD4/CD8比例较健康对照组明显减少（*t*分别为5.47、4.26，*P*分别为0.027、0.049），而晚期肺癌患者CD8^+^T细胞百分比较健康对照组增高，但差异无统计学意义（*t*=0.02, *P*=0.886）。此外，15例晚期肺癌患者CD4^+^CD25^+^T细胞百分比及CD4^+^CD25^+^FOXP3^+^T细胞百分比明显高于健康对照组（*t*值分别为5.17、5.93，*P*分别为0.034、0.024），结果见图 3A、B、C。

**3:**
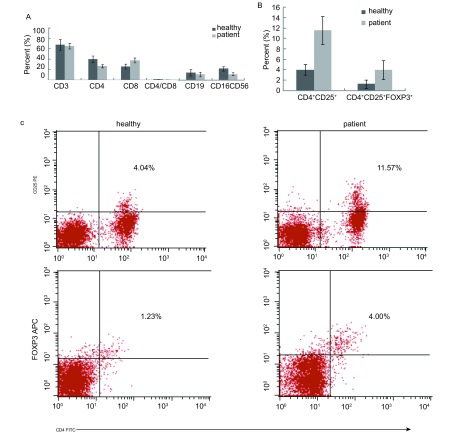
晚期肺癌患者和健康人外周血T、B、NK和调节性T细胞比例的比较A：T、B、NK细胞; B：调节性T细胞; C：调节性T细胞流式细胞仪检测结果。 Comparisons of the cell immunity between advanced stage of lung cancer patients and healthy individu

### 2.4 细胞内IFN-γ和IL-4阳性率的比较

10例晚期肺癌患者CD8^+^T细胞内细胞因子IFN-γ阳性率显著低于健康对照组，而患者IL-4阳性率明显高于健康对照组（*t*分别为5.03、7.34，*P*分别为0.038、0.014），结果见图 4A、图 4B。

**4:**
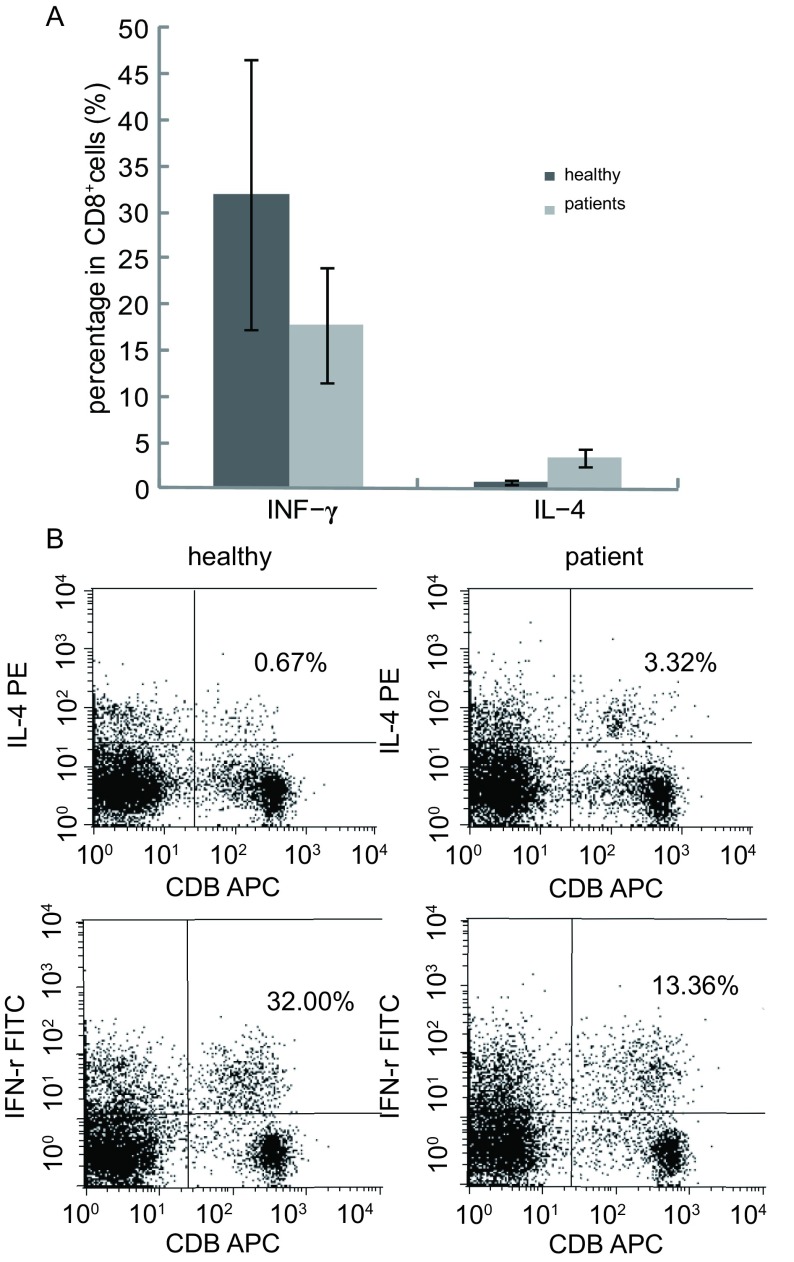
健康人及晚期肺癌患者细胞因子水平测定结果 Comparisons of the cytokine between advanced stage of lung cancer patients and healthy individuals

### 2.5 细胞内穿孔素及颗粒酶阳性率的比较

10例晚期肺癌患者外周血CD8^+^T细胞内穿孔素及颗粒酶阳性率均显著低于健康对照组（*t*分别为5.27、9.14，*P*分别为0.041、0.011），结果见图 5A、图 5B。

**5:**
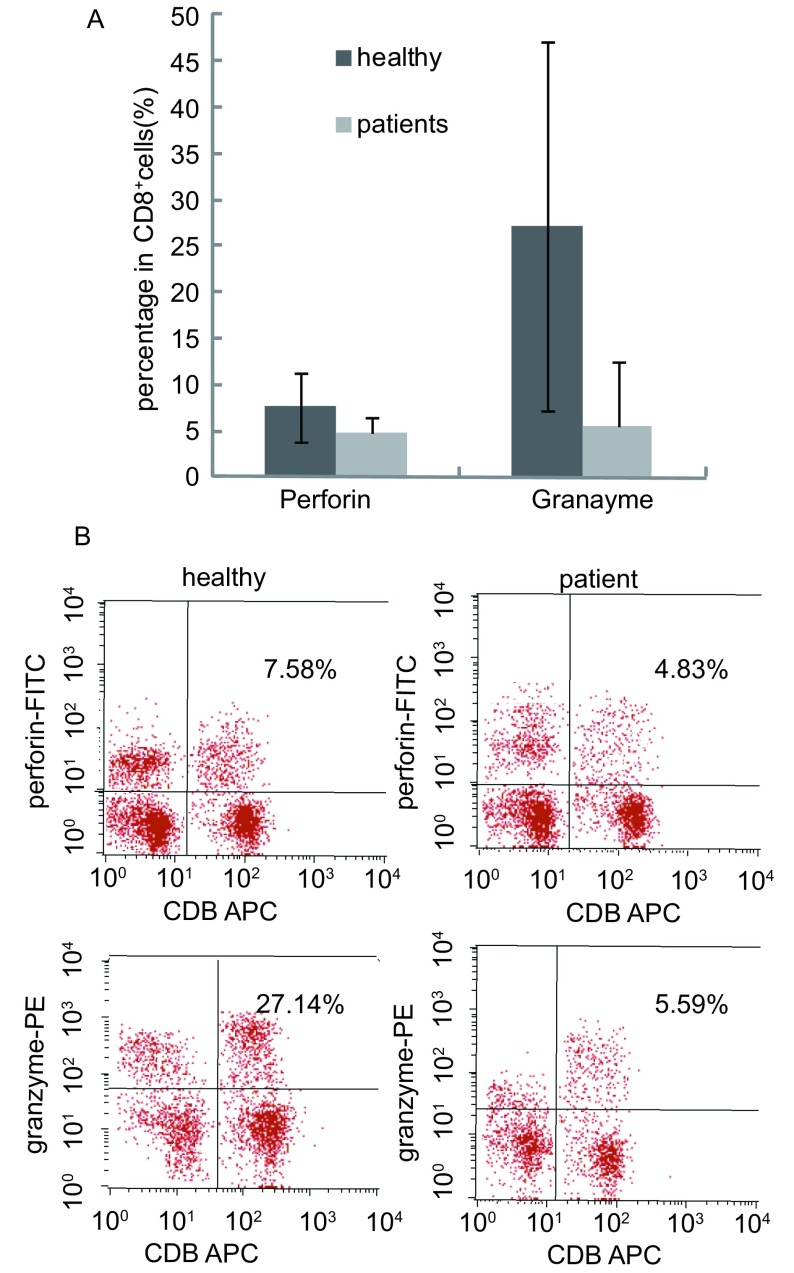
正常人及晚期肺癌患者细胞内穿孔素及颗粒酶比率的测定结果 Comparisons of perforin and granzyme levels in serum between advanced stage of lung cancer patients and healthy individua

### 2.6 免疫细胞体外细胞毒性比较

10例晚期肺癌患者外周血单个核细胞对A549及GM823细胞增殖的平均抑制率分别为（13.92±1.48）%、（12.43±1.17）%，显著低于健康对照组（27.49±5.42）%、（25.69±2.97）%（*P* < 0.05）。

### 2.7 免疫细胞体内细胞毒性比较

20例晚期肺癌患者PPD实验中，有16例为阴性，2例弱阳性，2例阳性; 而健康对照组中有2例为阴性，7例弱阳性，两者比较*P* < 0.01。

### 2.8 免疫细胞细胞增殖能力的比较

10例晚期肺癌患者外周血单个核细胞体外连续培养4天，从第1天始，细胞增殖能力均显著低于健康对照组（*P*分别为0.041、0.028、0.045、0.046），结果见图 6。

**6:**
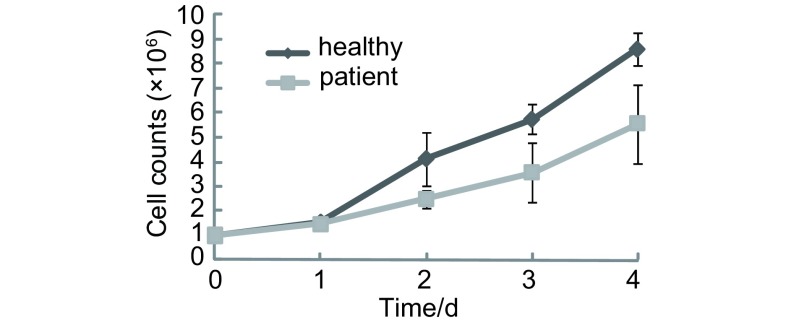
晚期肺癌患者及健康人外周血单个核细胞的增殖曲线 The growth curve of the mononuclear cells in peripheral blood between advanced stage of lung cancer patients and healthy individuals

## 3 讨论

一般认为， NK细胞和T细胞亚群在肿瘤免疫监视中起着重要的作用，其中CD3^+^/CD4^+^细胞被认为是机体免 疫系统中重要的细胞亚群， CD4/CD8比值被视为监视细胞免疫功能、反映机体免疫状态的重要指标^[4]^。本研究晚期肺癌患者外周血中免疫细胞绝对数显著低于健康对 照组，外周血CD3^+^、 CD3^+^/CD4^+^、 CD3^-^/CD16^+^56^+^T细胞 百分比及CD4/CD8比值较健康对照组明显降低，提示晚 期肺癌患者的免疫细胞明显减少。为了明确免疫细胞功 能，本实验对晚期肺癌患者及健康对照组进行体外细胞毒性试验及增殖试验，结果显示，患者免疫细胞对瘤细 胞株的抑制率及细胞增殖能力均低于健康对照组。而且晚期肺癌患者PPD试验阳性率明显低于健康组，进一步 说明肺癌晚期病人免疫细胞功能低下。同时，患者外周血中主要效应细胞CD8^+^T产生的穿孔素、颗粒酶阳性细 胞百分比明显低于健康组，进一步在分子水平说明了肿瘤晚期机体免疫功能低下的原因。

随着肿瘤免疫研究的深入，发现肿瘤在发生发展过程中并非被动过程，而是通过释放一些因子，诱发骨髓产生免疫抑制细胞^[5]^。CD4^+^CD25^+^T细胞是近年发现的在生理和病理状态下均具有重要免疫调节作用的抑制性T细胞亚群，许多研究显示，在多种肿瘤患者的血液中明显升高，且与肿瘤的病理类型、临床分期和预后有关^[6-8]^，是目前认为反映肿瘤疾病程度和疗效的良好指标。本研究结果显示，晚期肺癌患者CD4^+^CD25^+^Treg细胞及CD4^+^/CD25^+^/FOXP3^+^Treg占CD4^+^T细胞比例均明显高于健康对照组，提示晚期肺癌患者免疫功能失调与其比例增加有关。

肿瘤免疫抑制可通过抑制抗原提呈细胞的功能，继而抑制T细胞的功能发挥，改变Th细胞因子的分泌谱，使Th1型细胞因子（IFN-γ、TNF-γ、IL-12和IL-2等）分泌水平下降，Th2型细胞因子（IL-10和IL-4等）分泌水平上升，即由Th1向Th2的漂移，从而诱导Treg（CD4^+^/CD25^+^/FOXP3^+^）细胞的产生，并增强其抑制功能，发生免疫逃逸^[9]^。本研究检测结果显示，IFN-γ在晚期肺癌组的表达明显低于健康对照组，而IL-4相反，提示肺癌患者外周血CD8^+^T淋巴细胞向Th1细胞的分化明显减少，而Th2细胞明显增多，产生了由Th1向Th2的漂移。有研究认为，肺癌组织本身通过局部分泌或全身作用于外周血单个核细胞、肿瘤浸润性淋巴细胞，使其向Th2转化，导致机体处于免疫抑制状态，这可能是肿瘤细胞的主动性逃避和对抗机体免疫的机制之一^[10]^。

随着单克隆抗体在临床上的广泛应用^[11-13]^，体液免疫的抗肿瘤作用需再认识。近几年研究发现肿瘤发展的早期阶段B细胞可以通过使CD4^+^T细胞失活，导致细胞毒性T细胞（cytotoxic T lymphocyte, CTL）免疫功能降低，从而促进肿瘤的生长^[14]^。有研究表明，紫外线可以通过激活淋巴结内B细胞来抑制树突状细胞的功能，从而介导肿瘤免疫耐受^[15]^。但是，近几年的研究对肿瘤晚期患者B细胞是否也有免疫抑制作用结果不一致^[16, 17]^，有待于进一步探讨。本实验患者组CD19^+^B细胞数明显低于健康对照组，原因尚不清楚。我们以往研究^[18]^及本研究中均发现肿瘤患者IgG、IgA升高，是一种抗肿瘤反应？亦或是封闭性抗体？还是其它原因尚不明确，有待于进一步研究。但结合本研究中患者组补体C3、C4明显高于健康对照组，或许提示即使在晚期肺癌病人中，IgG等升高可能是通过ADCC作用发挥抗肿瘤作用。

总之，晚期肺癌患者无论从数量、功能上，还是体外杀瘤活性、增殖能力、体内PPD反应以及免疫抑制细胞、分子水平上均反映免疫功能低下。但是，本研究未能动态检测免疫功能演进状态，为以后研究的方向。

## References

[1] Serafini P, Borrello I, Bronte V (2006). Myeloid suppressor cells in cancer: Recruitment, phenotype, properties and mechanisms of immune suppression. Semin Cancer Biol.

[2] Ofran Y, Ritz J (2008). Targets of tumor immunity after allogeneic hematopoietic stem cell transplantation. Clin Cancer Res.

[3] Santomasso BD, Roberts WK, Thomas A (2007). A T cell receptor associated with naturally occurring human tumor immunity. Proc Natl Acad Sci USA.

[4] Bose A, Chakraborty T, Chakraborty K (2008). Dysregulation in immune functions is reflected in tumor cell cytotoxicity by peripheral blood mononuclear cells from head and neck squamous cell carcinoma patients. Cancer Immun.

[5] Gajewski TF, Meng Y, Harlin H (2006). Immune suppression in the tumor Microenvironment. J Immunother.

[6] Allan SE, Song-Zhao GX, Abraham T (2008). Inducible reprogramming of human T cells into Treg cells by a conditionally active form of FOXP3. Eur J Immunol.

[7] Gupta S (2008). Immune homeostasis: regulatory T cells (Treg) and molecules. J Clin Immunol.

[8] Ebert LM, Tan BS, Browning J (2008). The regulatory T cell associated transcription factor FoxP3 is expressed by tumor cells. Cancer Res.

[9] Hokey DA, Larregina AT, Erdos G (2005). Tumor cell loaded type-1 polarized dendritic cells induce Th1-mediated tumor immunity. Cancer Res.

[10] Cham CM, Gajewski TF (2005). Metabolic mechanisms of tumor resistance to T cell effector function. Immunol Res.

[11] Grothey A, Sugrue MM, Purdie DM (2008). Bevacizumab beyond first progression is associated with prolonged overall survival inmetastatic colorectal cancer: results from a large observational cohort study (BRiTE). J Clin Oncol.

[12] Bu P, Gao L, Zhuang J (2006). Anti-CD146 monoclonal antibody AA98 inhibits angiogenesis via suppression of nuclear factor kappa B activation. Mol Cancer Ther.

[13] Naka T, Iwahashi M, Nakamura M (2008). Tumor vaccine therapy against recrudescent tumor using dendritic cells simultaneously transfected with tumor RNA and granulocyte macrophage colonystimulating factor RNA. Cancer Sci.

[14] Byrne SN, Ahmed J, Halliday GM (2005). Ultraviolet B but not Aradiation activates suppressor B cells in draining lymph nodes. Photochem Photobiol.

[15] Matsumura Y, Byrne SN, Nghiem DX (2006). A role for inflammatory mediators in the induction of immunoregulatory B cells. J Immunol.

[16] Bracci L, Moschella F, Sestili P (2007). Cyclophosphamide enhances the anti-antitumor efficacy of adoptively transferred immune cells through the induction of cytokine expression, B-cell and T-cell homeostatic proliferation, and specific tumor infiltration. Clin Cancer Res.

[17] Varela JC, Imai M, Atkinson C (2008). Modulation of protective T cell immunity by complement inhibitor expression on tumor cells. Cancer Res.

[18] Li Y, Wang SY, Yu H (2007). Comparison of immune function between before and after chemotherapy of patients with malignant tumors and immune regulatory function of drugs. Chin J Cancer Prev Treat.

